# Assessment of in vitro particle dosimetry models at the single cell and particle level by scanning electron microscopy

**DOI:** 10.1186/s12951-018-0426-2

**Published:** 2018-12-07

**Authors:** Thomas Kowoll, Susanne Fritsch-Decker, Silvia Diabaté, Gerd Ulrich Nienhaus, Dagmar Gerthsen, Carsten Weiss

**Affiliations:** 1 Laboratory for Electron Microscopy, Karlsruhe Institute of Technology (KIT), Campus South, Engesserstr. 7, 76131 Karlsruhe, Germany; 2 Institute of Toxicology and Genetics, Karlsruhe Institute of Technology (KIT), Campus North, Hermann-von-Helmholtz-Platz 1, 76344 Eggenstein-Leopoldshafen, Germany; 3 Institute of Applied Physics, Karlsruhe Institute of Technology (KIT), Campus South, Wolfgang-Gaede-Str. 1, 76131 Karlsruhe, Germany; 4 Institute of Nanotechnology, Karlsruhe Institute of Technology (KIT), Campus North, Hermann-von-Helmholtz-Platz 1, 76344 Eggenstein-Leopoldshafen, Germany; 5 Department of Physics, University of Illinois at Urbana-Champaign, Urbana, IL 61801 USA

**Keywords:** Nanoparticles, FIB/SEM, Particokinetic models, Cellular dose

## Abstract

**Background:**

Particokinetic models are important to predict the effective cellular dose, which is key to understanding the interactions of particles with biological systems. For the reliable establishment of dose–response curves in, e.g., the field of pharmacology and toxicology, mostly the In vitro Sedimentation, Diffusion and Dosimetry (ISDD) and Distorted Grid (DG) models have been employed. Here, we used high resolution scanning electron microscopy to quantify deposited numbers of particles on cellular and intercellular surfaces and compare experimental findings with results predicted by the ISDD and DG models.

**Results:**

Exposure of human lung epithelial A549 cells to various concentrations of differently sized silica particles (100, 200 and 500 nm) revealed a remarkably higher dose deposited on intercellular regions compared to cellular surfaces. The ISDD and DG models correctly predicted the areal densities of particles in the intercellular space when a high adsorption (“stickiness”) to the surface was emulated. In contrast, the lower dose on cells was accurately inferred by the DG model in the case of “non-sticky” boundary conditions. Finally, the presence of cells seemed to enhance particle deposition, as aerial densities on cell-free substrates were clearly reduced.

**Conclusions:**

Our results further validate the use of particokinetic models but also demonstrate their limitations, specifically, with respect to the spatial distribution of particles on heterogeneous surfaces. Consideration of surface properties with respect to adhesion and desorption should advance modelling approaches to ultimately predict the cellular dose with higher precision.

**Electronic supplementary material:**

The online version of this article (10.1186/s12951-018-0426-2) contains supplementary material, which is available to authorized users.

## Background

Micro- and nanoparticles are increasingly used as additives in food and items of daily use, and they are also promising as tools for biotechnology and medicine. For the development of efficient as well as safe novel (bio)materials, a thorough understanding of their interactions with biological systems is required. Quantitative assessment of dose–response relationships is key to designing effective drug delivery systems or diagnostic agents, but also to assessing potentially adverse effects of small particles [1, 2]. To this end, in vitro studies are frequently used, in which cells are exposed to small particles suspended in cell-culture medium (CCM). In most cases, dosimetry is based on the nominal dose, i.e., the particle concentration applied in CCM, although not all particles will directly interact with the target cells. Therefore, Teeguarden et al. [3] suggested a more appropriate definition of dose in in vitro studies: the delivered, or cellular dose. Different metrics are used i.e. the mass, number or surface area of deposited particles. The delivered dose is highly dependent on a range of particle properties (e.g. size, shape, material density, agglomeration state, surface chemistry, surface charge) and characteristics of the CCM (density, viscosity). Gravitational settling dominates for larger particles whereas diffusion is relevant for small particles. Between 10 and 100 nm both processes equally impact particle transport [3, 4]. These physicochemical processes have profound effects on the deposited dose available for cellular interactions [5]. Whereas only a minor fraction of small, diffusing nanoparticles come in contact with cells, essentially all of the applied larger particles are finally deposited on the cell surface from where they may subsequently be internalized.

Various methods are routinely applied to determine the particle dose. For example, techniques for chemical bulk analyses (e.g. inductively coupled mass spectroscopy) are usually destructive and require dissolution of the biological specimen and particles. The delivered particle dose is calculated from these measurements but the information on the spatial localization of particles in the sample is completely lost. Thus, the distinction between the amount of particles on cell surfaces and internalized particles is impossible. Also, the problem of particle dissolution inside a cell or organism cannot be addressed and, therefore, the question of whether an intended effect or toxicity is initiated by the particles themselves or rather by their dissolved components, e.g., metal ions, remains unanswered. In contrast to the absolute quantification of particles by bulk analysis, light and electron microscopy enables detection at the single particle and cell level, yet at the cost of lower sample size. As a consequence, an urgent need for novel physical and chemical techniques exists to allow precise quantification of particle doses that simultaneously allow localization with high spatial resolution at the cell and tissue level.

Due to the difficulties in experimentally determining the delivered dose, Hinterliter et al. [4] were the first to develop a computational model named ISDD (In vitro Sedimentation, Diffusion and Dosimetry model) to calculate sedimentation, diffusion and the delivered dose. The widely accepted ISDD model has been further developed and takes into account the properties of the particles including agglomeration and dissolution, but it still has limitations [6–8]. Particles are removed from the virtual system once they have reached the bottom, which generates an additional concentration gradient enhancing diffusion to the bottom of the well. This leads to correct predictions in the case of strong particle adhesion at the outer cell membrane and/or fast uptake into cells. However, a different situation prevails for particles with only weak adhesion and/or slow cellular incorporation. Such particles may desorb from the outer cell membrane, so that the dose of particles interacting with cells is reduced.

To take different adhesive properties between cells and particles into account, DeLoid et al. [9] introduced the Distorted Grid (DG) model with an integrated “stickiness” parameter, which facilitates the simulation of surfaces with different adsorption strengths. The particle-cell surface interaction is emulated by a Langmuir binding isotherm [10], employing a user-defined equilibrium dissociation coefficient, K_D_. Reasonable values for K_D_ are in the order of 10^−8^ to 10^−9^ mol/L, which are characteristic for high-affinity binding typical of specific protein interactions [9]. Smaller K_D_ values relate to higher adsorption strength and therefore resemble more “sticky” boundary conditions and vice versa. Completely “non-sticky” boundary conditions disregard the influence of K_D_ on the concentration of free particles at the bottom of the suspension column. Each particle reaching the bottom remains unbound and, therefore, has the capability to diffuse back into upper layers. In this extreme scenario, the cell layer would resemble a reflective surface. As the probability of a particle to adhere to the cell layer is neither 0 (reflective conditions) nor 1 (complete stickiness), modelling relies on user-defined assumptions for an ill-defined K_D_. So far, no methods have been developed to directly determine the relevant K_D_ in different systems, which will depend on particle surface properties, and also on the cell type under investigation [11].

Here, we choose colloidal silica particles (SiO_2_) with nominal diameters of 100, 200 and 500 nm and A549 human alveolar epithelial cells as a widely used model system to assess the predictive power of different particokinetic models. Low particle concentrations were chosen to investigate more realistic delivered doses and to ensure that cells are not overloaded with excessive amounts of particles, which might artificially trigger toxicity [12, 13]. For the detection of such low particle numbers techniques with very high sensitivity and resolution are required. Therefore, scanning electron microscopy (SEM) was used to quantitatively determine the delivered dose, i.e., the number density of particles per area, denoted areal density (AD). The area in question is composed of the cellular surface area as well as the uncovered surface area of the cell culture dish (glass substrate) both of which presumably exhibit different adhesion properties. Hence, particle interactions with variable substrates but also cell surfaces is heavily influenced by multiple poorly specified parameters, with impact on the deposited dose, and have not been considered in computational models. Due to the high spatial resolution of SEM, we clearly show that the deposited particle ADs on cells are smaller than those in intercellular substrate regions. Interestingly, in the absence of cells, deposition of particles appears to be reduced, as substantially lower ADs are observed. Coating of the substrate by conditioned cell culture medium enhances the delivered particle dose, suggesting that cells facilitate adherence of particles to the substrate. Finally, we compared measured and calculated ADs using the DG and ISDD models. Whereas the ISDD model, assuming high absorbance of particles, predicts the experimental results for the deposited dose on the substrate (intercellular region) correctly, the DG model in the case of low stickiness more accurately captures the measured ADs on the cell surface.

## Results

### Particle characterization

Amorphous, spherical SiO_2_ particles with nominal diameters of 100, 200 and 500 nm and a mass density of 2 g/cm^3^ were used in this study. Particle sizes D_SEM_ were measured by SEM; they only slightly deviated from the nominal ones (roughly within 10%, cf. Table 1). In the following, we will refer to the nominal particle size to specify different measurements.

**Table 1:** Physico-chemical characterization of particles

SiO_2_ particle	D_SEM_ (nm)	D_DLS_ (nm) (0 h, RT)		ζ-Potential (mV)
		CCM	H_2_O	
100 nm NP	90 ± 8	173 ± 5	112 ± 2	− 29 ± 1
200 nm MP	188 ± 12	251 ± 6	188 ± 3	− 22 ± 1
500 nm MP	437 ± 23	532 ± 70	491 ± 17	− 40 ± 1

Nominal diameters are 100, 200 and 500 nm, as provided by the supplier. Diameter as observed by scanning electron microscopy (D_SEM_), hydrodynamic diameter (D_DLS_), in cell culture medium (CCM) and water (H_2_O), and ζ-potential

*NP* nanoparticle, *MP* microparticle, *DLS* dynamic light scattering, *SEM* scanning electron microscopy

We assessed the stability of the working suspensions and the ζ-potentials by using dynamic light scattering (DLS). Furthermore, hydrodynamic diameters D_DLS_ were measured by DLS because they are needed for modelling the fate and transport of particles in suspension. The results of the particle characterizations are compiled in Table 1 and Additional file 1, and do no indicate any severe agglomeration after preparation of suspensions. Hence, the nominal particle density was sufficient for simulation purposes; there was no need to determine effective densities of agglomerates.

However, the D_DLS_ values of all particles in cell culture medium (CCM) were somewhat higher (1.9-, 1.3- and 1.2-fold increase for the 100, 200 and 500 nm particles, respectively) than the D_SEM_ values. According to Nienhaus et al. [14] the layer thickness for most proteins adsorbed at the surface of nanoparticles varies between 3 and 7 nm, depending on the specific protein investigated. Thus the protein corona on particles suspended in DMEM supplemented with fetal bovine serum (FBS) should increase the particle diameter by about twice the protein extension. Furthermore, the electrochemical double layer determining the final hydrodynamic diameter can be estimated by the Debye length in CCM, which can be calculated to approximately 1 nm for an ionic strength of 0.13 mol/L [15]. Therefore, assuming an average thickness of 5 nm for the protein corona, the particle diameter in complete CCM would increase by around 12 nm. One could argue that the larger hydrodynamic diameters are due to the formation of small agglomerates of 2 to 3 particles in CCM, but a thorough in vitro analysis of the deposited particles by SEM showed that approximately 90% of the 100 and 200 nm particles found on the substrates are single particles. Hence, we conclude that the particle suspension is for the most part monodisperse and the seemingly larger hydrodynamic diameters are due to lacking accuracy of the DLS measurements in complete CCM.

For 500 nm particles, only approximately 60% of all deposited particles appeared as single entities. The remaining 40% were detected as dimers, trimers or even bigger agglomerates. As the scattering of light is proportional to the sixth power of the diameter, these agglomerates, if they would have been formed already in the suspension, are supposed to scatter the light at least 64 times stronger than the single particles, resulting in a pronounced peak in the size range of 1 to 2 µm. Since we do not observe such a peak but one broad peak at around 530 nm (cf. Additional file 1a), we conclude that the working suspension of 500 nm particles was monodisperse, too, and agglomerates or rather accumulates are formed after deposition on the substrate surface.

By contrast, DLS measurements in H_2_O yielded diameters more similar to those obtained by SEM (D_DLS_ in H_2_O, cf. Table 1 and Additional file 1b). For simulations with the DG model, we therefore used these D_DLS_ values and added twice the average thickness of a protein corona of 5 nm.

### Cellular and intercellular measurements of particle areal densities

In vitro experiments were performed to measure the delivered particle dose. A549 cells were cultured on indium-tin-oxide (ITO)-coated glass substrates (cf. “Materials and methods” for details). ITO-coated substrates are particularly well suited for SEM measurements because charging artefacts are avoided by the electrically conducting ITO film. Different ADs were obtained by varying exposure times and nominal particle concentrations for the three different particle sizes.

**Fig. 1:**
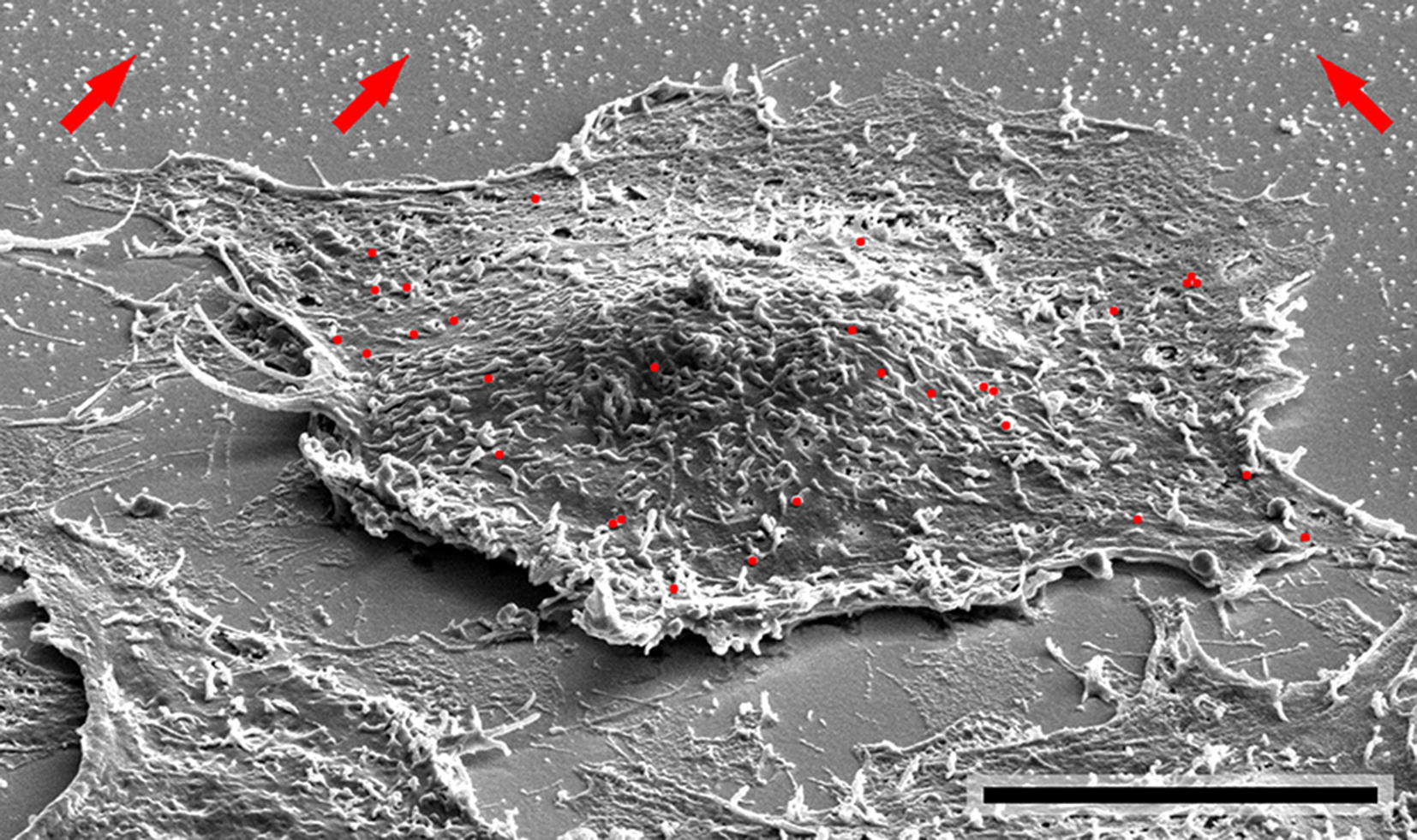
Representative 3 keV SE SEM image of A549 cells and intercellular regions after deposition of 100 nm particles for 4 h. The image was acquired at a tilt angle of 52°. Particles on top of a selected cell are marked in red for better visibility. Arrows indicate particles in intercellular regions. *SE* secondary electrons. Scale bar: 10 µm

The representative small-magnification secondary electron (SE) SEM image in Fig. 1 shows substrate regions without cells and cells after deposition of 100 nm particles for 4 h. Regions with different ADs are distinguishable. A high particle concentration is observed on the substrate at some distance from the cells. These regions will be denoted “intercellular regions” in the following. The concentration of particles on the cells (marked by red dots for better visibility) is substantially lower. This also applies to the substrate regions close to the cells, where particles are essentially absent. The low particle concentration in these regions is attributed to the shrinkage of cells due to the preparation process for SEM. Therefore, ADs in close proximities to cells were not considered further.

**Fig. 2:**
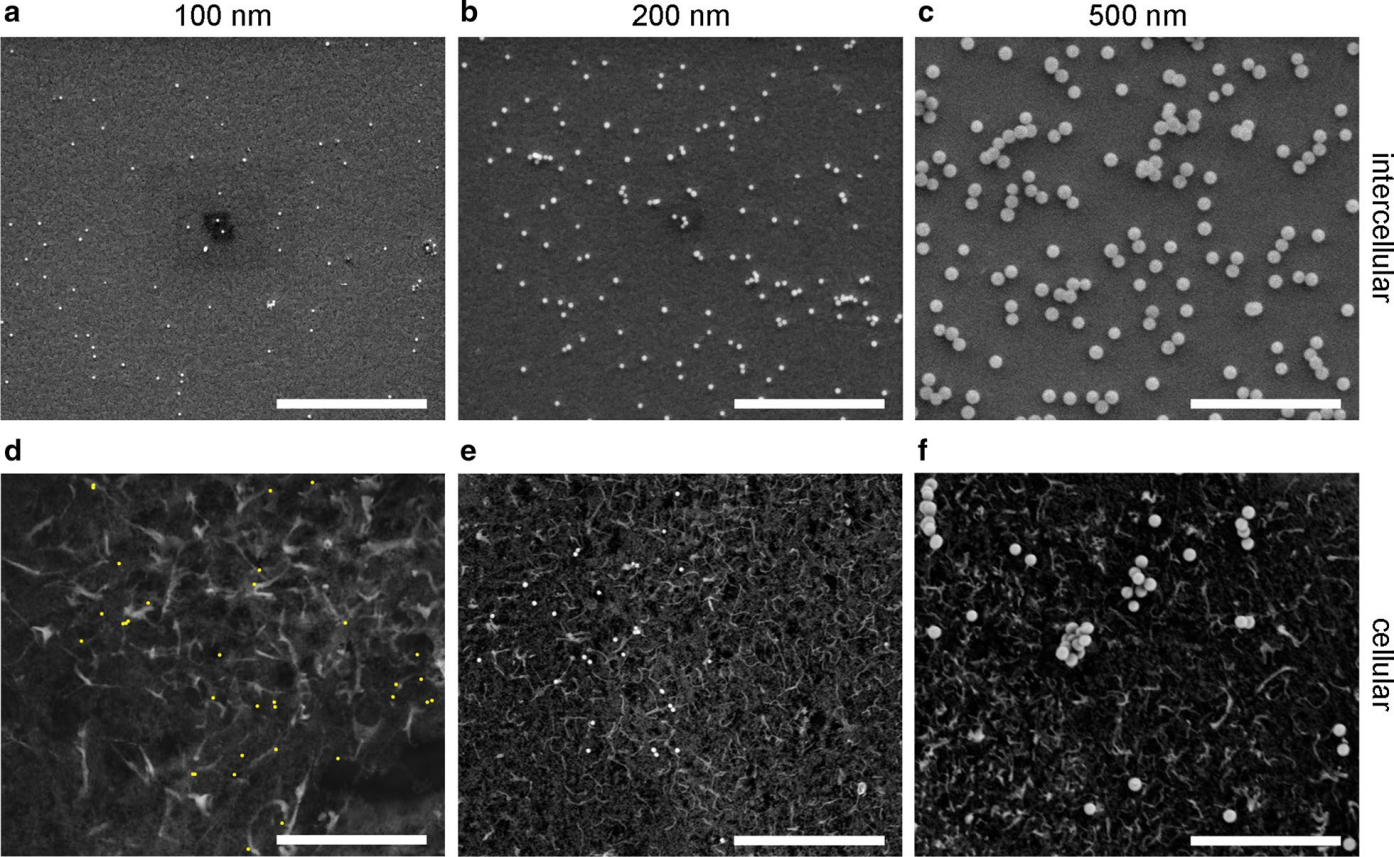
Representative SEM images illustrating different intercellular (**a**–**c**) and cellular (**d**–**f**) ADs after deposition of differently sized particles for 1 h. **a**, **d** 100 nm particles with input particle concentration of 7 µg/mL, **b**, **e** 200 nm particles with input concentration of 50 µg/mL and **c**, **f** 500 nm particles with input particle concentration of 109 µg/mL. Due to their small size, 100 nm particles in **d** are marked in yellow for better visualization. **a**–**c** SE SEM images were taken with 5 keV electron energy. **d**–**f** BSE SEM images were taken with 5 keV electron energy and a deceleration voltage of 1.9 kV (**a**) and 4 kV (**e**, **f**). Small dark rectangles are electron beam induced perturbations from previous scans. Scale bar: 5 μm

SEM images of intercellular regions (Fig. 2a–c) and the corresponding SEM images of the cell surfaces (Fig. 2d–f) from the corresponding samples show representative average ADs for three different experiments yielding the deposition of 100 nm (Fig. 2a, d), 200 nm (Fig. 2b, e) and 500 nm particles (Fig. 2c, f). We note that composition-sensitive backscattered electron (BSE) SEM images were taken to improve the particle contrast on the cell surface because particles are more difficult to detect in SE SEM images. Further, we would like to point to the strong topographical contrast of cellular structures such as filopodia, which appear as white, elongated structures and should not be misinterpreted as silica particles. Visual image inspection demonstrates that the ADs are different in intercellular and cellular regions for all particle sizes. Furthermore, ADs are rather homogeneously distributed across various fields of view on ITO/glass substrates, whereas inhomogeneous patterns of ADs are found in cellular regions. For example, in Fig. 2e, a high AD is found for 200 nm particles in the left half of the image, whereas particles are almost completely absent in the right half.

**Fig. 3:**
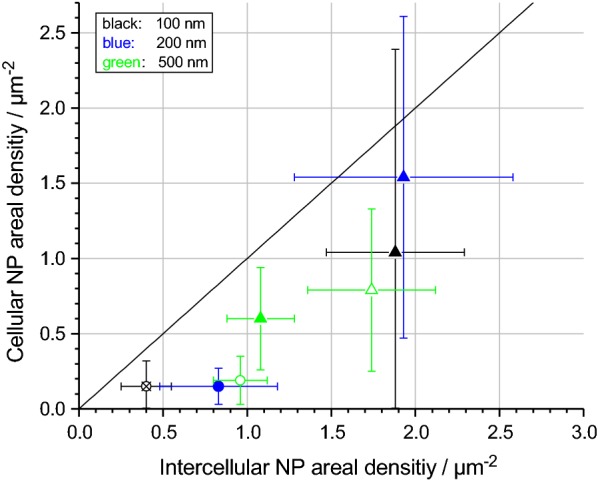
Comparison of measured particle ADs on A549 cell surfaces and in intercellular regions. Deposition experiments were performed for 100 nm (black), 200 nm (blue) and 500 nm (green) silica particles with different input concentrations for 1 h (circles) and 4 h (triangles) incubation time. Full symbols denote 50 µg/mL input concentration, empty symbols 109 µg/mL and crossed symbols 7 µg/mL. The diagonal line marks hypothetical identical cellular and intercellular ADs. *AD* areal density

The measured ADs are summarized in Fig. 3, where data from intercellular and cellular regions are compared. Figure 3 demonstrates that the AD is always lower on cell surfaces compared to intercellular regions. For example, the average intercellular AD of 100 nm particles after 1 h of exposure is 0.40 ± 0.15 µm^−2^ (cf. Fig. 2b), whereas it is only 0.15 ± 0.15 µm^−2^ on cells (cf. Fig. 2e). The large statistical errors result from the variability of average local ADs over macroscopic sample regions. This is particularly the case for cellular measurements (variations up to 130%) because the deposition on cells is more heterogeneous, as described above.

Particle uptake in cells must be considered as an explanation for lower ADs in cellular regions. To investigate the particle concentration within cells, cells were cut open slice-by-slice by focused-ion-beam (FIB) milling and simultaneous SEM imaging. A short video sequence composed of a series of SE SEM images illustrating the slicing and imaging process is included in Additional file 2. Several 200 nm particles are visible on the cell surface in the video sequence, whereas none are found inside the cell. FIB/SEM imaging demonstrates that particle concentrations in cells are negligible for all particle sizes. FIB/SEM results are confirmed by scanning transmission electron microscopy investigations of thin sections of embedded A549 cells (cf. Additional file 3).

According to the FIB results, particle uptake can be clearly ruled out as a reason for the large discrepancies between cellular and intercellular ADs. Differences in adhesion of particles to cells and intercellular regions most likely explain the divergent ADs.

We have chosen early time points (1 and 4 h) to clearly address the deposition of particles on and aside cells. Notably, we have excluded later time points, at which particles are taken up by cells and the intercellular space has vanished because the entire surface is covered by cells due to cell division. Furthermore, we selected particles which are non-toxic under the specified conditions. Cellular toxicity often complicates quantitative analysis of particle deposition and uptake as cells disintegrate and the cellular content including particles are released and distributed. This is actually also the reason why cellular imaging or quantification of the cellular particle dose of toxic particles is often performed at early time points, i.e., before cellular disintegration becomes apparent, to obtain some meaningful data [16, 17].

Nevertheless, we investigated a single later time point, i.e., 24 h, which is often used in cell culture experiments (cf. Additional files 4 and 5). For a better comparison with the 1 and 4 h time points, we selected particle concentrations which should yield similar deposited numbers of particles per µm^2^. As expected, cells covered almost the entire surface due to cell division (see Additional file 5a) and the ADs of 100 nm and 200 nm particles at the cellular surface and in the few remaining intercellular regions were drastically reduced in comparison to earlier time points (cf. Additional file 4). Therefore, cellular processes such as uptake of particles by A549 cells reported previously [18, 19] but also cell density at later time points reduce the detectable deposited dose on and adjacent to cells, respectively. Interestingly, for the larger 500 nm particles, the calculated numbers accurately predicted the experimentally validated numbers; no major difference could be observed (Additional files 4 and 5). As A549 cells are epithelial and not phagocytic cells, uptake of particles is mediated by endocytosis [18, 19] which has an upper size limit that is exceeded by the 500 nm particles [20]. So far, dosimetry models do not account for these important biological processes and thus are limited to only predict the deposited dose impacted by physicochemical forces. Hence, future work in the field of particokinetics needs to incorporate such aspects of cell biology and expand the existing quantitative models.

### Influence of substrate coatings on areal densities of particles

To further elucidate the adhesion of particles on different surfaces, cell-free deposition experiments were performed by applying different precoatings. The first set of experiments was performed in the absence of cells. The ITO/glass-substrates were incubated in CCM (supplemented with serum) before being exposed to silica particles. Additionally, in a second set of experiments, the substrates were pre-exposed to so-called conditioned CCM, which represents the supernatant of cultivated A549 cells and contains, e.g., additional secreted proteins (cf. “Materials and methods” for details).

**Fig. 4:**
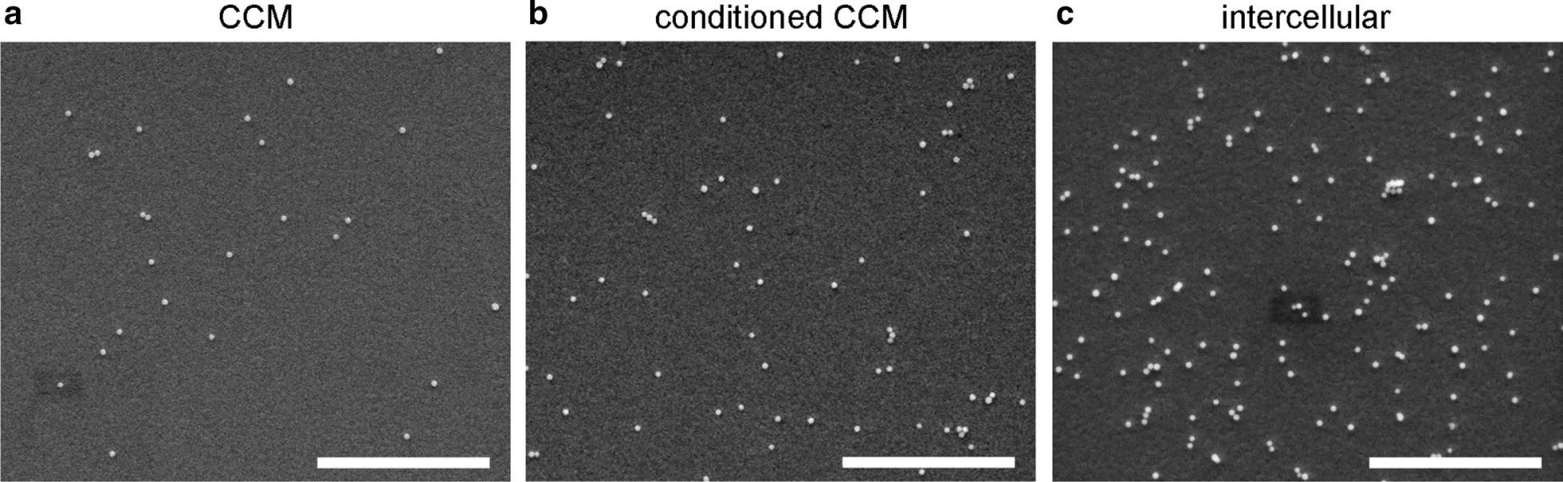
Representative SEM images illustrating the impact of different surface coatings of ITO/glass substrates on ADs of particles. All experiments were performed with 200 nm silica particles with an input concentration of 50 µg/mL and 1 h incubation time for **a**, **b** without cells and **c** with cells. **a** Precoating with CCM, **b** precoating with conditioned CCM derived from A549 cell cultures and **c** intercellular region of an ITO/glass substrate overgrown with A549 cells. Small dark rectangles are electron beam induced perturbations. *CCM* cell culture medium. Scale bar: 5μm

Figure 4 shows representative SEM images of ADs after 1 h deposition of 200 nm particles with the same particle input concentration (50 µg/mL) in the absence (Fig. 4a, b) or presence of cells (Fig. 4c). Particle ADs are significantly smaller in the absence of cells compared to the AD in intercellular regions of the corresponding experiment with cells. Precoating with conditioned CCM (Fig. 4b) slightly increases the AD compared to precoating with pure CCM (Fig. 4a), but the presence of cells clearly yields the highest AD (Fig. 4c).

**Fig. 5:**
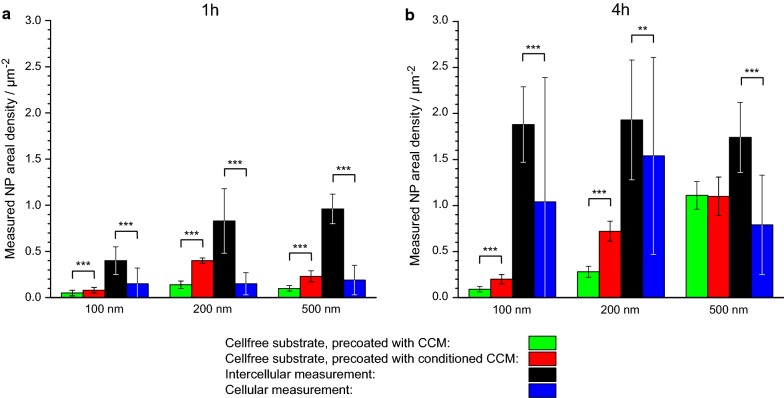
Bar charts summarizing the effects of different substrate coatings on average ADs. ADs were determined on substrates in the absence of cells, which were either pre-coated with CCM or conditioned CCM. In parallel, intercellular and cellular ADs in the presence of A549 cells are depicted. Different particles sizes (100, 200, and 500 nm) and incubation times were assessed as indicated. Input concentrations were 7 µg/mL (100 nm), 50 µg/mL (200 nm) and 109 µg/mL (500 nm), except for the deposition of 100 nm NPs for 4 h in the presence of cells (50 µg/mL). Despite partly large individual errors, t-tests show a high statistical significance in most cases (p-values indicated by stars above bars: **< 0.01, ***< 0.001)

In addition to the qualitative observations outlined above, we performed quantitative analyses of the different deposited dose of particles dependent on the precoating of the substrate but also in relation to cellular and intercellular regions (Fig. 5). ADs on the CCM-precoated substrates without cells (green bars) are lower than on those precoated with conditioned CCM (red bars). This effect is observed at 1 and 4 h for all particle sizes (100-, 200- and 500 nm) except for the 4 h time point for 500 nm particles. As deposition for the largest particles is mainly driven by sedimentation, the influence of the adhesive properties of the substrate seems to be less important, especially at later time points. In intercellular regions (black bars), the ADs are indeed, as suggested above (Fig. 4), maximal and again much greater than in the regions covered by cells (blue bars).

### Comparison of measurements with simulations

**Fig. 6:**
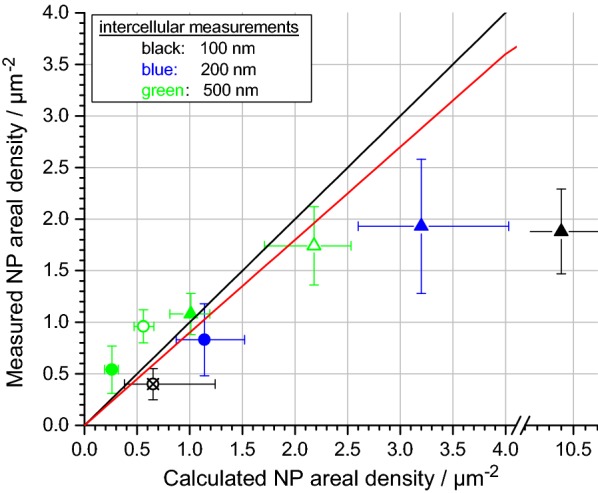
Measured intercellular ADs compared with calculated ADs using sticky boundary conditions (K_D_ = 10^−9^ mol/L). ITO/glass substrates covered with A549 cells were incubated with 100 nm (black), 200 nm (blue) and 500 nm (green) SiO_2_ particles at different concentrations for 1 h (circles) and 4 h (triangles). Full symbols denote 50 µg/mL input concentration, empty symbols 109 µg/mL and crossed symbols 7 µg/mL. Note the interrupted x-axis between 3.5 and 10.0 µm^−2^. The black diagonal line indicates an ideal match between measured and calculated ADs. The red diagonal line displays the result of linear regression analysis with fixed intercept at zero, excluding a single outlier (black, full triangle)

The measured intercellular ADs are compared with simulations performed with the DG model in Fig. 6. Simulations were performed by assuming “sticky” boundary conditions with K_D_ = 10^−9^ mol/L, because high ADs were found under these conditions. Further simulation parameters are listed in Additional file 11 (cf. “Materials and methods”). The simulations are in good agreement with the measurements, apart from one extreme outlier for 100 nm particles und 4 h deposition time (full black triangle, note the interrupted x-axis). A linear regression analysis (diagonal red line), excluding the mentioned outlier, yielded a slope of 0.9 with a Pearson correlation coefficient of 0.93, which confirms the tight correlation. A possible reason for the much lower measured values in case of the 100 nm particles at 4 h might be the saturation of available binding sites at the surface, an effect which the model does not consider and, therefore, it predicts higher ADs. We note that the calculated ADs of the DG model agree with the results from ISDD, because ISDD innately uses “sticky” boundary conditions (cf. Additional file 6). For comparison, simulations with “non-sticky” boundary conditions were also performed (cf. Additional file 7). Indeed, the DG model emulating a non-sticky surface clearly underrates the deposited ADs, resulting in poor agreement of the measured and simulated deposited dose.

**Fig. 7:**
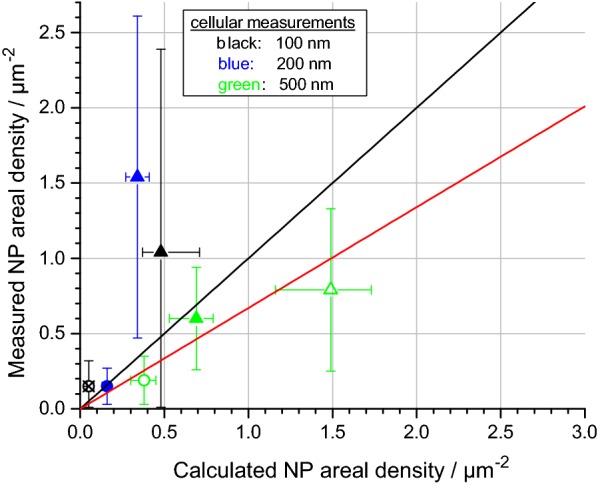
Measured cellular ADs compared with calculated ADs for non-sticky boundary conditions. ITO/glass substrates covered with A549 cells were incubated with 100 nm (black), 200 nm (blue) and 500 nm (green) silica particles at different concentrations for 1 h (circles) and 4 h (triangles). Full symbols denote 50 µg/mL input concentration, empty symbols 109 µg/mL and crossed symbols 7 µg/mL. The black diagonal line indicates an ideal match between measured and calculated ADs. The red diagonal line displays the result of linear regression with fixed intercept at zero

Next, we compared the measured and calculated ADs on A549 cells (Fig. 7). A “non-sticky” surface was assumed for the simulations, because measured ADs on cells are lower compared to intercellular ADs (cf. Fig. 5). A reasonable agreement is obtained in all cases, as also evidenced by linear regression analysis (slope: 0.67; Pearson’s r: 0.87). In contrast, in case of “sticky” boundary conditions, the DG model drastically overestimates the really deposited dose (cf. Additional file 8).

In summary, adhesion of particles to the cell surface of A549 cells seems to be very weak, whereas their interactions with the ITO/glass surface appear to be much stronger. Therefore, considering differences in K_D_ during modelling allows proper prediction of the deposited dose on inhomogeneous surfaces comprised of cellular and intercellular regions. Such comparisons at cellular resolution have not been performed so far in the context of computational models and highlight the need to integrate spatial information on surface properties to reliably quantify the local deposition of particles.

**Fig. 8:**
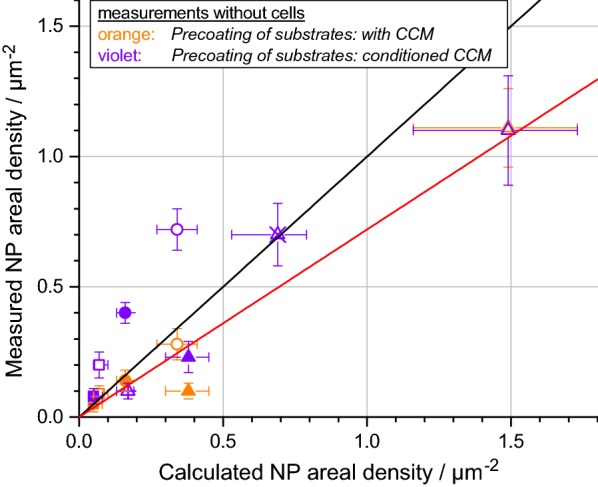
Measured ADs on cell-free pre-coated substrates compared with calculated ADs using non-sticky boundary conditions. Deposition experiments were performed for cell-free ITO/glass substrates, pre-coated with CCM or conditioned CCM with 100 nm (squares), 200 nm (circles) and 500 nm (triangles) silica particles at different concentrations for 1 h (full symbols) and 4 h (empty symbols). Orange color represents pre-coatings performed with CCM and violet color represents pre-coatings with conditioned CCM. The black diagonal line indicates an ideal match between measured and calculated ADs. The red diagonal line displays the result of linear regression analysis with fixed intercept at zero

Finally, we analyzed the deposition of particles onto ITO/glass substrates in the absence of cells (cell-free substrates, Fig. 8). When the substrate was precoated either with CCM or conditioned CCM, the DG model applying “non-sticky” boundary conditions correctly predicts the deposited dose as also corroborated by regression analysis (slope: 0.52, Pearson r: 0.88). However, when assuming strong adhesion to the surface, the correlation between measured and calculated ADs is very poor (cf. Additional file 9). This quantitative comparison suggests that, although precoating of ITO/glass surfaces with CCM promotes particle adhesion to some extent, which is further improved by addition of conditioned CCM (Fig. 4), these surfaces are weakly adhering surfaces. In the presence of cells, however, the ITO/glass surfaces in the intercellular regions show much stronger adhesion, as suggested by computational modelling.

## Discussion

A heterogeneous deposition of the delivered particle dose in in vitro experiments was observed, resulting in substantial variations of the ADs. Most notably, the dose on cell surfaces was distinctly lower than on intercellular areas. Simulations of ADs with the DG model demonstrate that weak particle adsorption to cellular membranes, i.e., “non-sticky” boundary conditions, must be assumed to achieve agreement between measured and calculated ADs. Furthermore, deposition of particles onto ITO/glass substrates precoated with CCM or conditioned CCM was significantly reduced with respect to measurements in the presence of cells. Therefore, cells seem to promote adsorption of particles in the intercellular space. In conclusion, existing particokinetic models are well suited to predict the particle dose on homogeneous surfaces, specifically, if adsorptive properties are adjusted according to experimental validation. However, as the rate constants of adsorption and desorption on different cellular and intercellular surfaces are ill-defined, the spatiotemporal distribution of particles as well as the cellular dose still need to be experimentally validated and quantified by appropriate methods.

Our results show, that SEM is an excellent technique for this purpose. Due to its high sensitivity and spatial resolution very small delivered doses, down to a few particles per 100 µm^−2^, were successfully quantified. At the same time, sufficient statistical power was achieved, as *t*-tests of the obtained data showed. Semi-automatic counting of particles by our recently developed software facilitates image analysis (“Materials and methods”). The striking difference in particle numbers on cells and in the intercellular regions was further analyzed. First, we investigated cellular uptake of particles by STEM and FIB/SEM, techniques successfully used to quantify number of particles per cell at the highest possible resolution [21, 22]. In line with studies with bronchial epithelial cells (BEAS-2B), we could not detect substantial amounts of silica particles in A549 cells [23]. The efficiency of uptake depends on various parameters, e.g., cell type, physicochemical properties of particles and the presence of a protein corona. Indeed, precoating of silica particles with serum proteins has been shown to reduce particle uptake and toxicity [17, 18, 24–28]. Lesniak et al. [28] demonstrated that lipid bilayers rapidly adsorb proteins from complete cell culture medium, which hinders adsorption of silica particles covered by a protein corona, whereas bare particles strongly adhered. Most likely, the low adsorption of protein-coated silica particles onto the membrane of A549 cells prevents efficient endocytosis, as suggested previously [19]. Interestingly, in the absence of a protein corona, binding of silica particles to A549 cells is drastically enhanced. Considering the high diffusion constants of the silica particles (e.g. approximately 5 µm^2^/s for 100 nm silica particles), the particles move around rapidly and are immobilized once they hit an adsorptive surface. As A549 cells appear as poorly adhering surfaces, possibly due to the lack of dedicated receptors [29], protein-coated silica particles rather encounter sites in the intercellular region with higher adsorptive properties. The exact nature of such adhesion sites is presently unknown. The ITO/glass substrate is presumably covered by proteins derived from the cell culture medium and hence is unlikely to interact strongly with the protein-coated silica particles. As conditioned cell culture medium and, even more, cultivation of cells enhances particle adsorption onto the ITO/glass substrate, it is tempting to speculate that cells secrete factors which promote adhesion. Degradation of the protein layer by, e.g., secreted proteases might foster interaction of the bare ITO/glass surface with the protein-coated particles. The impact of cells on particle deposition is not without precedence. Albanese et al. [30] showed that incubation of gold nanoparticles with conditioned medium enhances particle aggregation and changes the protein corona. Both factors were suspected to contribute to increased cell membrane adhesion, uptake and retention of nanoparticles. The incorporation of cell-secreted proteins into the protein corona might increase the affinity of particles to selected receptors or even support binding of particles to additional receptors. Similar observations were made by Rischitor et al. [31], who found that deposition of gold nanoparticles is strongly dependent on the presence or absence of A549 cells. Only a fraction of particles deposited on a cell monolayer was detected on polystyrene wells in the absence of cells.

For predicting the deposited dose, mostly the ISDD and DG models are employed. In line with our findings, it has been shown that both models predict the same ADs assuming sticky boundary conditions, meaning that particles adhere strongly to the surface [11]. However, in the case of weakly adhering surfaces, the DG model is better able to estimate deposition kinetics, which was experimentally validated by comparing calculated and quantified concentration profiles in frozen sections of columns of nanoparticle suspensions [9]. However, verification of the ISDD and DG models at the single cell and particle level has not yet been performed [11]. Our SEM studies indicate that the ISDD and DG models correctly predict the deposited amount of silica particles for intercellular regions when emulating a “sticky” surface. In contrast, the DG model using “non-sticky” boundary conditions is better suited to calculate ADs on cells which do not efficiently interact with or incorporate particles. In the future, there is a clear need to quantify adsorption and desorption across heterogeneous surfaces to further improve particokinetic models and, in the end, to more accurately predict the cellular dose. High resolution optical microscopy in real time at the single particle level allows one, in principle, to measure different rate constants for various labeled particles and different substrates as well as cell types. Both single particle tracking [32] as well as super-resolution imaging [33] approaches have been shown to be feasible. Still, these techniques are challenging and have not yet found wide-spread application.

Current modelling is not only limited by our inability to properly evaluate the spatiotemporal distribution of particles on heterogeneous surfaces, but also by the inaccuracy of particle size determination in complex media, which profoundly influences the predicted dose. Uncertainties in the hydrodynamic diameters drastically affect calculated ADs. The smaller the particles, the more sensitive is the calculated dose to variations of D_DLS_. For example, varying the hydrodynamic diameter of 500 nm particles by ± 52 nm leads to errors of approximately 10%, whereas a ± 22 nm variation of the hydrodynamic diameter of 100 nm particles leads to errors between 40 and 90% (sticky boundary conditions assumed). Therefore, considering the broad particle size distribution in cell culture medium, we took here the mean hydrodynamic diameter in water and added another 10 nm to account for the protein corona as an estimate of the overall size. This approach resulted in very good agreement of measured and calculated ADs. This points to the compelling necessity of precisely knowing the hydrodynamic diameter for meaningful calculations. Probably, other techniques, e.g., fluorescence correlation spectroscopy [34], should also be employed to measure particle size in suspension to more rigourously address the validity of the selected method in the context of modelling the deposited dose.

Finally, some advantages and limitations of SEM as a technique to study particle distribution and dosimetry should be considered. The lateral spatial image resolution of SEM is theoretically about 1 nm and the thickness of the consecutive FIB slices can be decreased to a few ten nanometer, as stated by Guehrs et al. [22]. So, also smaller NPs can be analyzed by SEM and FIB-SEM bearing in mind that resolution is improved with atoms of higher atomic number. Thus, e.g., gold NPs are easier to detect than silica NPs. In practice, scanning electron microscopy can resolve single NPs down to 3 nm if they contain materials with high atomic number such as gold [38]. In our own previous studies, also platinum NPs with a size of about 100 nm were detected by FIB-SEM as individual NPs inside colon carcinoma cells [39]. In a recent FIB-SEM study [22] with silver NPs of similar size (75 nm) and exposure dose (10 μg/cm^3^), roughly 3000 NPs could be detected in a single monocyte. Theoretically, there is no upper limit for the detection of intracellular NPs as long as they can be identified as single entities. However, dependent on the morphology of NPs or, in the case of severe agglomeration, separation of single NPs might prove to be challenging and, hence, quantification of absolute numbers, specifically at high concentrations, could become infeasible.

Quantitative analysis by electron microscopy is extremely time-consuming and laborious. Therefore, more data points were not included, e.g., addressing variations due to different cell types or surface chemistry and charge of the NPs. Clearly, this is a major limitation of the technique. Whereas, for example, image acquisition and quantitative analysis for NP exposed cells by conventional fluorescence microscopy can be performed within a few seconds per image covering hundreds of cells [41], sample preparation and imaging by SEM coupled with semi-automatic NP quantification takes up to a week, and only a few cells can be investigated. Therefore, SEM cannot be applied as a high-throughput technique but is rather suited to support quantitative studies in selected cases. This is especially relevant for quantitative evaluation of NP uptake, which most often relies on destructive methods such as inductively coupled plasma mass spectrometry (ICP-MS) [5]. Our approach of detailed analysis of NP quantification and localization on and adjacent to cells will provide spatio-temporal information to further improve our understanding of particle-cell interactions.

## Conclusions

Particokinetic models are important tools to predict the cellular dose. Although such models have been further developed over the last years and improved to also include agglomeration, dissolution and adherence of particles, the spatiotemporal distribution on surfaces has not been addressed in detail. Our finding of a heterogeneous deposition dependent on the “stickiness” of the cellular and intercellular surface has major implications for the accurate calculation of the delivered cellular dose and, thus, for the proper establishment of dose–response curves. Hence, for the reliable determination of toxicity as well as pharmacological efficiency of various particles or particulate systems, a verification of the number of deposited particles is still required, preferably by using high-resolution techniques such as SEM. As a perspective, quantification of particle adherence and desorption could enable the modeling of such crucial surface interactions to determine the effective cellular dose in a more reliable fashion.

## Materials and methods

### Materials

Dulbecco’s modified Eagle’s medium (DMEM), fetal bovine serum (FBS), streptomycin, penicillin and Dulbecco’s phosphate buffered saline without calcium and magnesium chloride (DPBS^−/−^) were obtained from Life Technologies (Frankfurt, Germany). Paraformaldehyde (PFA) was purchased from Sigma-Aldrich (Taufkirchen, Germany). 6-Well plates were purchased from Greiner Bio-One (Nürtingen, Germany). Methanol was obtained from VWR International (Bruchsal, Germany). Ethanol was from Carl Roth (Karlsruhe, Germany). Monodisperse, amorphous SiO_2_ particles with nominal diameters of 100, 200, and 500 nm were purchased from Postnova Analytics (Landsberg am Lech, Germany). Indium tin oxide (ITO)-coated glass slides were obtained from PGO (Iserlohn, Germany).

### Cell culture

The human alveolar epithelial cell line (A549) was purchased from the American Type Culture Collection (ATCC Rockville, USA) and cultured as previously described by Panas et al. [35]. Cells were cultured on indium-tin-oxide (ITO) coated glass substrates [36], which are particularly well suited for SEM investigations of cells and deposited particles because charging artefacts during SEM image acquisition are minimized by the electrically conducting ITO film.

Conditioned CCM was prepared by culturing adherent growing A549 cells at a density of 1.3 × 10^4^ cm^−2^ for 72 h and centrifugation of the CCM supernatant at 300*g* for 5 min.

### Preparation of particle suspensions

Aqueous stock solutions of 100, 200 and 500 nm SiO_2_ particles (50 mg/mL) were vortexed for 10 s and diluted with deionised water to a final concentration of 1 mg/mL. After vortexing for additional 10 s, working suspensions were prepared by further dilution in CCM at the indicated concentrations and then added onto ITO-coated glass cover slips. In order to obtain roughly similar ADs irrespective of particle size, appropriate input concentrations were calculated using the DG model (see below). As a result, particle concentrations between 7 and 109 µg/mL depending on the particle size were applied. The resulting ADs up to maximally 2 particles per μm^2^ enabled accurate counting of individual particles, as numbers should neither be too low nor too high for SEM analysis. Too few particles per area would not yield statistically relevant data; a dense particle layer would render quantitative AD evaluation impossible. Furthermore, low concentrations of nanoparticles (NP) were intentionally deposited as realistic exposures of lung cells in humans even under extreme conditions are only up to 300 NPs per cell [12]. Thus, with a surface area of about 300 μm^2^ per cell, the chosen dose range of 0.1 to 1.5 NP/μm^2^ is in accordance with reasonable cellular doses and avoids excessive overload which can trigger adverse effects [13]. Indeed, conventional experiments on submerged cells often use much higher NP concentrations reaching doses of up to 10^8^ NPs per cell [12], which dramatically exceed realistic and physiologically relevant doses.

### Particle treatment

After cleaning ITO-coated glass cover slips in a nitrogen stream and thoroughly rinsing with 80% methanol/water (v/v), the as-prepared substrates were inserted into 6-well plates. For particle exposure under cell-free conditions, 3 mL of CCM was directly added to the ITO-coated substrates. Alternatively, for cellular experiments, A549 cells were seeded onto the ITO-coated glass slides in the 6-well plate at a density of 8 × 10^4^ cm^−2^ and allowed to attach overnight in 3 mL CCM. On the following day, the medium was removed and the ITO-coated surfaces were incubated with 3 mL of particle working suspension (see above) for 1 h and 4 h, respectively.

### Scanning electron microscopy (SEM)

To preserve the samples for SEM analysis and avoid loss of particles and cells, the substrates as well as the cells and particles were fixed by chemical cross-linking with freshly prepared 4% paraformaldehyde solution in DPBS^−/−^ (w/v) for 10 min at RT (room temperature) and washed three times with DPBS^−/−^. Afterwards, the samples were dehydrated in a graded ethanol series (50, 70, 95 and 100%, each concentration was applied twice in succession for 10 min each), followed by critical point drying. The dry specimens were placed on aluminium sample holders (Plano GmbH, Wetzlar, Germany) with a diameter of 32 mm for SEM investigations and contacted with conductive silver paint.

All samples were investigated with a Quanta 650 ESEM (FEI, Hillsboro, OR) equipped with an Everhart–Thornley detector for secondary electron (SE) imaging and a silicon solid-state backscattered electron (BSE) detector. Images were obtained from two particular regions of interest: cell-free regions between the cells (intercellular areas) and the cellular surfaces (cellular areas). Care was taken to avoid taking images in the direct vicinity of cells so that their natural movement during particle exposure but also possible shrinkage during sample preparation would not influence reliable quantification of intercellular particle ADs.

For intercellular areas, SE imaging mode at 5 keV primary electron (PE) energy was adequate, while cellular images were taken in BSE mode with 5 keV PE energy and a retarding bias voltage applied to the stage. Since the cellular surface has a pronounced topography, particles were hardly visible in SE imaging mode and, therefore, BSE imaging was employed to exploit its material contrast capability. A bias was needed to enhance the NP contrast, because the difference in average atomic number between SiO_2_ (${\bar{\text{Z}}}$ = 10) and the cell material, basically consisting of carbon (${\bar{\text{Z}}}$ ≈ 6), is very small. For 200 nm and 500 nm microparticles (MPs) the maximum bias voltage was − 4 kV, while for 100 nm NPs a reduced bias of − 1.9 kV was used to avoid detecting accelerated SEs, which might lead to small cellular structures being misinterpreted as NPs.

For statistics, series of 50 to 100 images of each sample and region of interest were taken and evaluated with respect to their particle ADs. Magnifications were chosen such that areas as large as possible could be depicted while maintaining proper particle visibility. We made an effort to cover a large substrate region to minimize the impact of local inhomogeneity. The error bars of overall measured ADs in Fig. 3 indicate the variation of local average ADs evaluated from single SEM images.

### Particle AD quantification

Particles in SEM images were quantified with the help of a semi-automatic routine written in Matlab (The MathWorks, Inc., Natick, MA), based on a template-based cross-correlation algorithm developed by Simon et al. [37]. In essence, it compares a SEM image with an averaged template of the particle in question and calculates the AD based on the area covered by the image and the number of particles detected. The program further allows us to inspect the result visually and to manually add missed particles or remove false positives (cf. Additional file 10). Therefore, the automated detection process can be ruled out as a possible source of error. Statistical significance of measured ADs was assessed by Student’s *t*-tests.

### Particle transport simulations

In this work, particle sedimentation was computed by the Distorted Grid (DG) fate and transport model by DeLoid et al. [9]. In this two-dimensional model, a particle suspension column is divided into even compartments. For each compartment, the change in concentration by diffusion and sedimentation is calculated as a function of time. A big advantage of the DG model is the implemented “stickiness” factor, K_D_, which allows one to simulate different adsorption strengths of the surface. K_D_ is the equilibrium dissociation constant of a Langmuir isotherm adsorption process [10] and is defined as

$$K_{D} = \frac{{\left( {1 - \theta } \right)\left[ P \right]}}{\theta }$$

where θ is the fraction of surface sites occupied, i.e., the fractional surface coverage, and [P] is the molar concentration of particles. Using K_D_, the fraction of particles bound to the bottom compartment can be calculated. These particles are then subtracted from the total particle concentration in the bottom compartment, resulting in the concentration of free particles in the bottom compartment. Only these free particles can possibly diffuse back to the compartment on top. Therefore, the free particle concentration is used to calculate the concentration changes by diffusion in the two lowest compartments. Typical K_D_ values range from 10^−8^ to 10^−9^ mol/L; smaller K_D_ values indicate a higher adsorption strength and therefore resemble more “sticky” surface conditions and vice versa.

In contrast, “non-sticky” conditions disregard the influence of K_D_ on the concentration of free particles at the bottom of the suspension column. Every particle reaching the bottom remains unbound and therefore has the capability to diffuse back into the upper layers. Thus, the bottom layer acts as a reflective surface. For a more detailed description of the implementation of “stickiness”, we refer to DeLoid et al. [9].

The DG model also allows us to consider broader particle size distributions as well as agglomerates. However, for the case of the silica particles investigated here, the size distribution was rather narrow and confined to only a single peak. Hence, the average hydrodynamic diameters derived from DLS measurements could be used. A complete overview of all parameters can be found in Additional file 11.

Uncertainties of derived parameters have to be taken into consideration when comparing calculated and measured ADs. Two main parameters were identified as possible sources of error for calculations: the hydrodynamic diameter and the concentration of the working suspensions. Because of inaccuracies during preparation of working suspensions, we estimated a 10% uncertainty on this parameter. In case of the hydrodynamic diameter, the difference between measured D_DLS_ values in H_2_O (plus 2 × corona thickness) and the theoretically expected values, given by D_SEM_ + 2 × corona thickness + 2 × Debye length in DMEM, was used as an estimate, except for the 200 nm particles. Because both D_DLS_ in H_2_O and D_SEM_ were identical, we again estimated a 10% error. By varying these two parameters in the DG model by their respective uncertainties, the impact on calculated ADs of each parameter’s uncertainty was derived. The overall error of calculated ADs was then computed as the root mean square of the individual errors.

### Dynamic light scattering (DLS)

DLS measurements for determining the hydrodynamic diameter of SiO_2_ particles in aqueous solutions were performed by using a Nanosizer Nano ZS (Malvern Instruments, Southborough, USA), as previously described [40].

### Focused ion beam (FIB)/SEM analysis

Combined FIB/SEM investigations were performed using a Strata 400S (FEI). Besides an electron column for SEM imaging, it provides a Ga^+^ ion column for ion milling, which is tilted 52° with respect to the electron column. This setup allows to mill perpendicular to the sample surface, while SEM images are obtained from 52° angle allowing a side view of the sample.

Samples had to be coated with a thin carbon layer beforehand, to improve the electrical conductivity of the surface to reduce charging artefacts while FIB milling. Single cells were randomly chosen and cut open slice-by-slice with 30 kV Ga^+^ ions. The step size was chosen to be 1/4 to 1/5 of the corresponding particle diameter presumably to be found in the cell, so that no particle would be accidentally removed from the cell undetected. After each step, a secondary electron (SE) SEM image at 10 keV PE energy was taken.

### Scanning transmission electron microscopy (STEM)

For STEM investigations a different preparation routine was necessary, which was performed as follows. After placing Transwell^®^ culture plate inserts (Corning, New York, NY) in 6-well plates, the lower chamber was filled with 2.5 mL CCM while A549 cells were seeded onto the membrane at a density of 3.7 × 10^5^ cm^−2^ in 1.5 mL CCM. The cells were allowed to attach overnight. On the next day, the medium in the upper chamber was removed and the cells were incubated with 1.5 mL of particle working suspension (see above) for 4 h. Afterwards, the medium in both chambers was removed and the cells were washed with 1 mL 0.1 M PIPES [piperazine-N,N′-bis(ethanesulfonic acid)] buffering agent. The insert was then placed in a petri dish. Samples were cut out using a biopsy punch and placed in 1.5 mL microcentrifuge tubes, where the first fixation using a fixative composed of paraformaldehyde and glutaraldehyde was performed overnight at 4 °C. Afterwards, the samples were washed twice for 10 min with 0.1 M PIPES, followed by the second fixation in an osmium tetroxide solution on ice for 1 h. Again, the samples were washed twice with 1 mL 0.1 M PIPES buffering agent and twice with water, for 15 min each. Subsequently the samples were stained overnight in 2% uranyl acetate dissolved in 25% ethanol/75% deionised water.

The fixed and stained samples were then dehydrated with a graded ethanol series (50, 70, 95 and 100%, each 2 times for 10 min), followed by two 5 min treatments in 100% propylene oxide. For embedding the samples in epoxy resin (EPON), graded series of EPON in propylene oxide (30, 70, 100% for 1 h, overnight, 6 h) were applied. Subsequently, the samples were placed in embedding moulds, doused with EPON solution and stored for 3 days at 60 °C for hardening. Finally thin slices were prepared using a Leica EM UC6 ultramicrotome and placed on TEM copper grids for STEM investigations, which were performed using a Strata 400S (FEI) at 30 keV PE energy. High-angle angular dark-field imaging mode was chosen due to its high material contrast. Images of whole cell cross sections as well as close-ups were obtained.

## Additional files

**Additional file 1.** Representative DLS size distributions of 100 nm, 200 nm and 500 nm SiO_2_ particles in CCM (a) and H_2_O (b). Measurements were performed immediately after suspensions were prepared at RT.

**Additional file 2.** Movie illustrating the slicing and viewing process used to investigate particle uptake by A549 cells. FIB milling was carried out perpendicular to the sample surface, while SEM imaging allows for a tilted cross-section view at an angle of 52°. After each milling step (step size 30–50 nm), an SE SEM image is obtained. The sequence of images is played as a movie.

**Additional file 3.** Representative STEM images of an A549 cell cross-section showing negligible uptake of 100 nm silica NPs. (a) A549 cell (centre) cultured on a Transwell^®^ membrane (bottom) which as a whole are embedded in EPON resin (top). Thin slices (thickness ≈ 100 nm) were prepared using ultramicrotomy and placed onto TEM grids. Throughout STEM investigations, only minor particle uptake was observed, though the differences between cellular and intercellular measurements suggested substantial uptake of particles. This representative cellular cross-section contains only one silica NP, which is marked with a green arrow in the enlarged segment (b). Dark rectangular regions on the images result from electron beam induced perturbations from previous scans.

**Additional file 4.** Tabular comparison of calculated ADs to measured intercellular and cellular ADs after deposition of 100 nm, 200 nm and 500 nm SiO_2_ particles for 24 h. On ITO/glass substrates growing A549 cells were exposed to 100 nm 200 nm and 500 nm SiO_2_ particles for 24 h and then prepared for SEM analysis. Intercellular and cellular ADs were measured from SEM images by counting deposited particles. 12–24 regions of interest (ROI) were evaluated for each treatment. n.d.: not detectable.

**Additional file 5.** Representative SEM images of A549 cells and intercellular regions after deposition of 500 nm SiO_2_ particles for 24 h. ITO/glass substrates covered with A549 cells were exposed to 25 µg/mL SiO_2_ particles with 500 nm diameter for 24 h (b–f). Control cells received CCM alone (a). Also note the strong adhesion of particles to the two mitotic cells in the lower right corner of panel (d). Scale bar: (a) 100 µm, (b-f) 10 µm.

**Additional file 6.** Comparison of calculated ADs using the DG model and ISDD. Using sticky boundary conditions within the DG model (green), almost identical values are obtained, whereas calculations with non-sticky boundary condition (blue) do not match the calculations with ISDD. The black diagonal line indicates an ideal match. The solid red line displays the result of linear regression analysis of the sticky (green) data with fixed intercept at zero (slope 1.01, Pearson correlation coefficient: 1.0), whereas the dashed red line displays the result of linear regression analysis of the non-sticky (blue) data with fixed intercept at zero (slope 0.07, Pearson correlation coefficient: 0.67).

**Additional file 7.** Measured intercellular ADs compared with calculated ADs using non-sticky boundary conditions. ITO/glass substrates covered with A549 cells were incubated with 100 nm (black), 200 nm (blue) and 500 nm (green) SiO_2_ particles at different concentrations for 1 h (circles) and 4 h (triangles). Full symbols denote 50 µg/mL input concentration, empty symbols 109 µg/mL and crossed symbols 7 µg/mL. The black diagonal line indicates an ideal match between measured and calculated ADs. The red line displays the result of linear regression analysis with fixed intercept at zero (slope 1.76, Pearson correlation coefficient: 0.87). Note the marked difference between the red and the black lines, indicating less agreement of measured and simulated results.

**Additional file 8.** Measured cellular ADs compared with calculated ADs using sticky boundary conditions (K_D_ = 10^−9^ mol/L). ITO/glass substrates covered with A549 cells were incubated with 100 nm (black), 200 nm (blue) and 500 nm (green) SiO_2_ particles at different concentrations for 1 h (circles) and 4 h (triangles). Full symbols denote 50 µg/mL input concentration, empty symbols 109 µg/mL and crossed symbols 7 µg/mL. The black diagonal line indicates an ideal match between measured and calculated ADs. The red line displays the result of linear regression analysis with fixed intercept at zero (slope 0.19, Pearson correlation coefficient: 0.82). Note the greatly differing slopes of the red and the black lines, indicating poor agreement of measured and simulated results.

**Additional file 9.** ADs measured on cell-free pre-coated substrates are compared with calculated ADs using sticky boundary conditions (K_D_ = 10^−9^ mol/L). Deposition experiments were performed with cell-free ITO/glass substrates, precoated with CCM and conditioned CCM with 100 nm (squares), 200 nm (circles) and 500 nm (triangles) silica particles at different concentrations for 1 h (full symbols) and 4 h (empty symbols). Orange color represents pre-coatings performed with CCM and violet color represents pre-coatings with conditioned CCM. The black diagonal line indicates an ideal match between measured and calculated ADs. The red line displays the result of linear regression with fixed intercept at zero (slope 0.15, Pearson correlation coefficient: 0.8). Note the difference in slope between the red and the black lines, indicating poor agreement of measured and simulated results.

**Additional file 10.** SE SEM image of 100 nm SiO_2_ NPs deposited on ITO substrate analysed with a semi-automated Matlab routine. The detected particles are counted and marked with colored circles, where green indicates accurate and red uncertain classification, which should be checked by the operator. The white arrow points at a small accumulation of NPs, of which only a small number has been detected, and the blue arrow shows a missed NP. After visual inspection, missing particles can be added, and false positives can be removed manually from the total sum of particles by the operator.

**Additional file 11.** Tabular summary of the parameters used with the DG fate and transport model for computing particle deposition.
