# Supplementary figures and images for: Assessment of in vitro particle dosimetry models at the single cell and particle level by scanning electron microscopy
Source: J Nanobiotechnology. 2018 Dec 7;16:100. doi: 10.1186/s12951-018-0426-2 (PMC6284276; doi:10.1186/s12951-018-0426-2)

a)

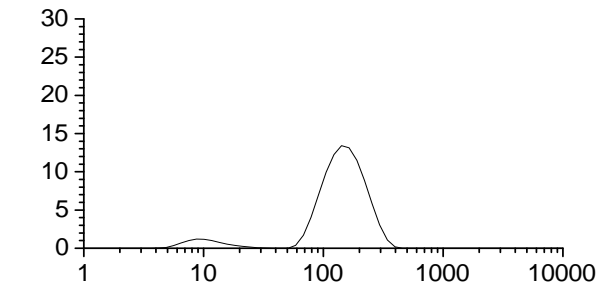

b)

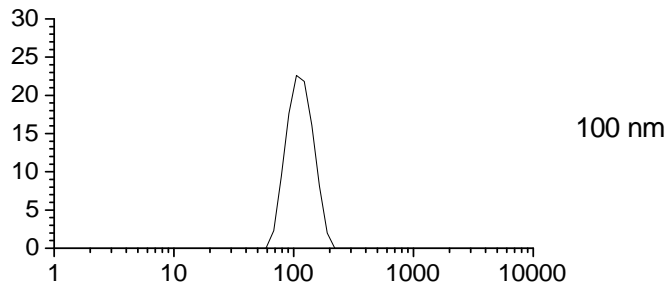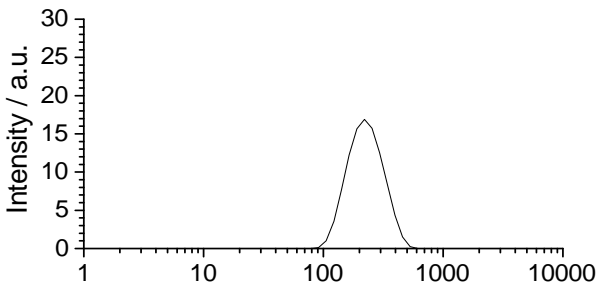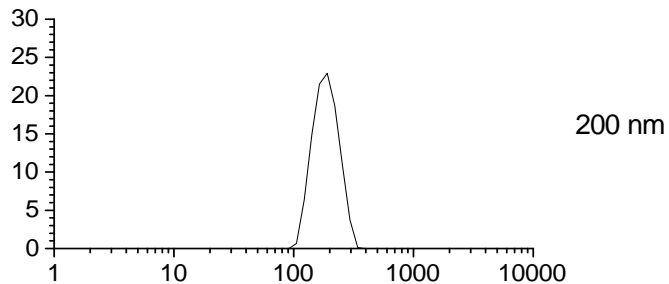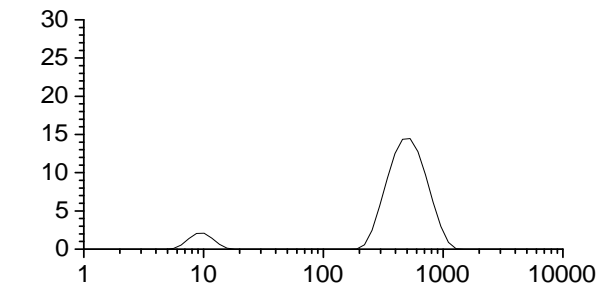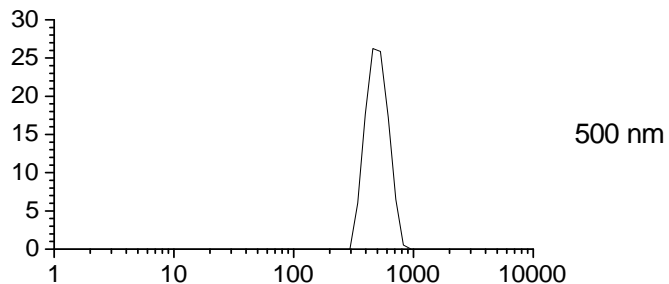

Size / nm

Supplement: Supplementary file 1 — Additional file 1. Representative DLS size distributions of 100 nm, 200 nm and 500 nm SiO2 particles in CCM (a) and H2O (b). Measurements were performed immediately after suspensions were prepared at RT. [file 12951_2018_426_MOESM1_ESM.pdf]

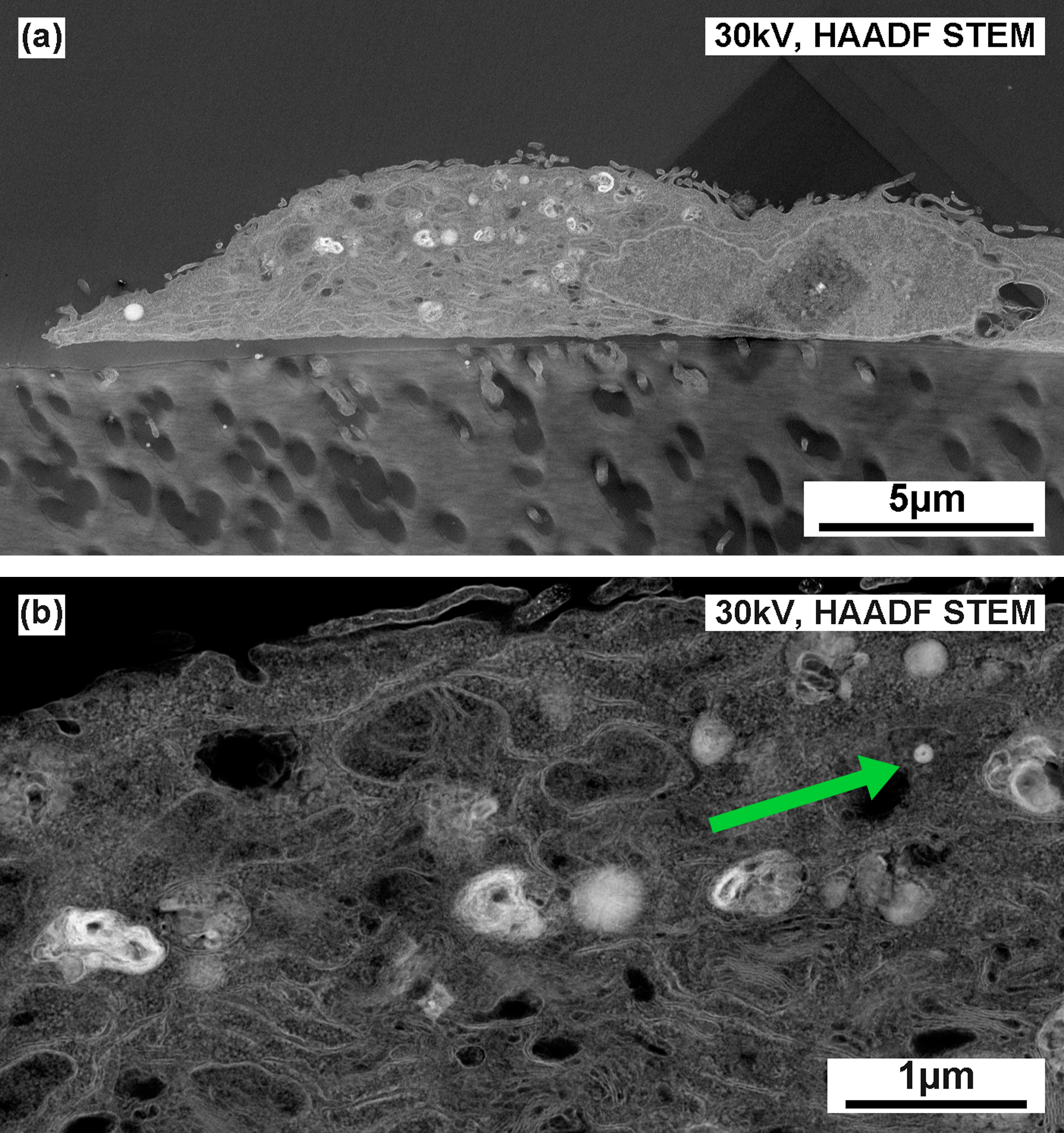

Supplement: Supplementary file 3 — Additional file 3. Representative STEM images of an A549 cell cross-section showing negligible uptake of 100 nm silica NPs. (a) A549 cell (centre) cultured on a Transwell® membrane (bottom) which as a whole are embedded in EPON resin (top). Thin slices (thickness ≈ 100 nm) were prepared using ultramicrotomy and placed onto TEM grids. Throughout STEM investigations, only minor particle uptake was observed, though the differences between cellular and intercellular measurements suggested substantial uptake of particles. This representative cellular cross-section contains only one silica NP, which is marked with a green arrow in the enlarged segment (b). Dark rectangular regions on the images result from electron beam induced perturbations from previous scans. [file 12951_2018_426_MOESM3_ESM.tif]

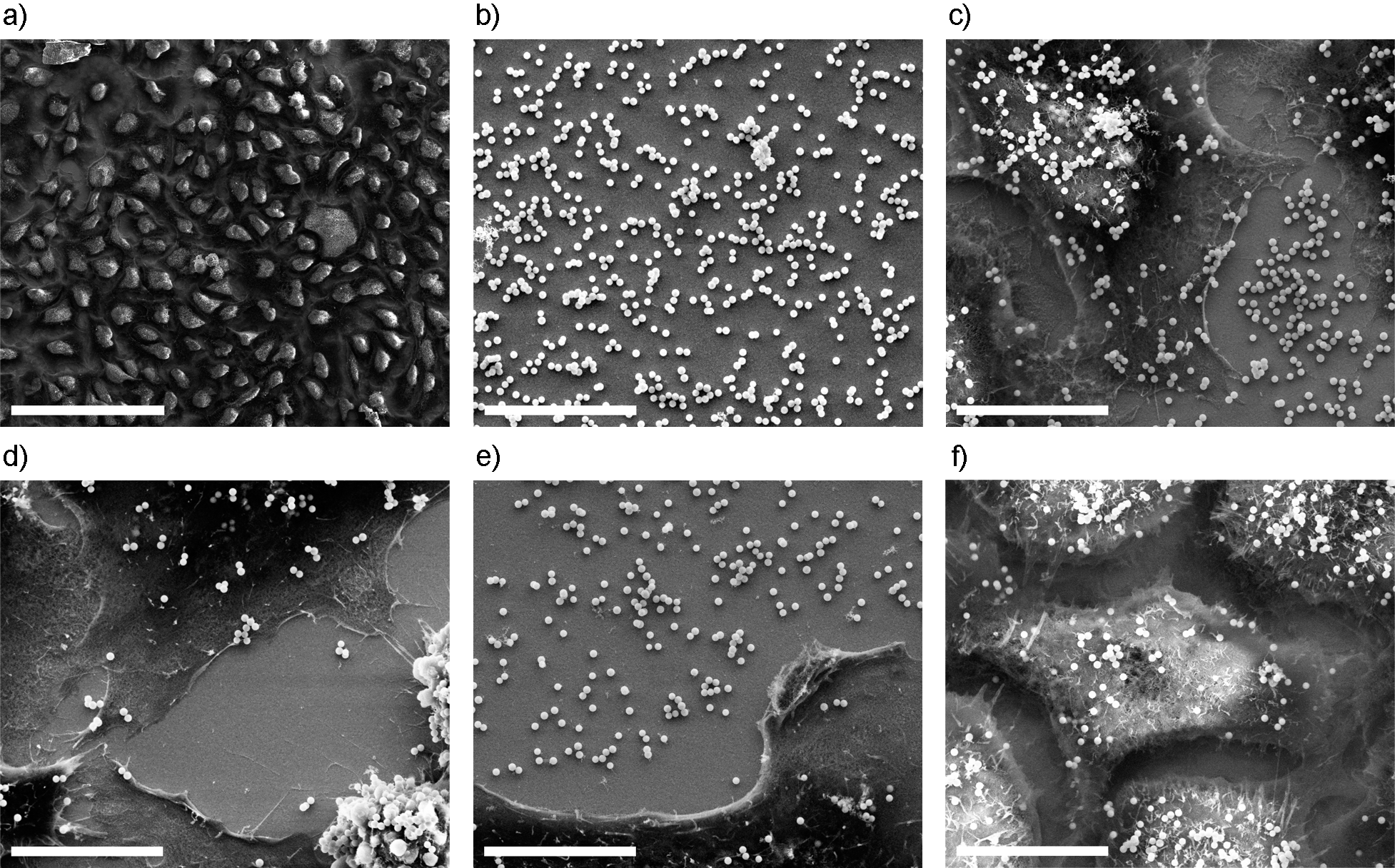

Supplement: Supplementary file 5 — Additional file 5. Representative SEM images of A549 cells and intercellular regions after deposition of 500 nm SiO2 particles for 24 h. ITO/glass substrates covered with A549 cells were exposed to 25 µg/mL SiO2 particles with 500 nm diameter for 24 h (b–f). Control cells received CCM alone (a). Also note the strong adhesion of particles to the two mitotic cells in the lower right corner of panel (d). Scale bar: (a) 100 µm, (b-f) 10 µm. [file 12951_2018_426_MOESM5_ESM.tif]

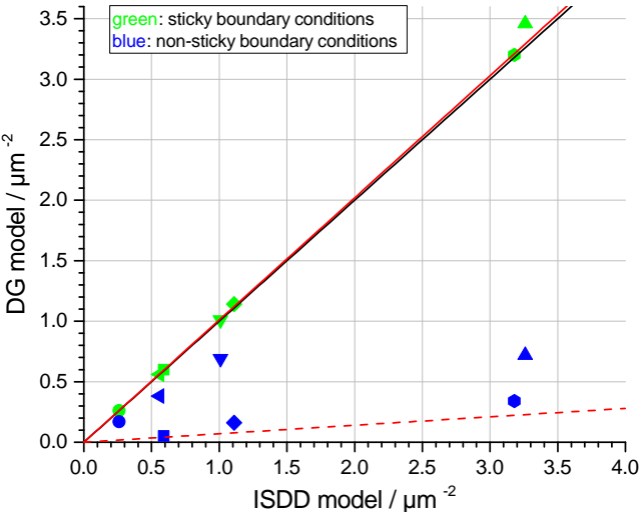

Supplement: Supplementary file 6 — Additional file 6. Comparison of calculated ADs using the DG model and ISDD. Using sticky boundary conditions within the DG model (green), almost identical values are obtained, whereas calculations with non-sticky boundary condition (blue) do not match the calculations with ISDD. The black diagonal line indicates an ideal match. The solid red line displays the result of linear regression analysis of the sticky (green) data with fixed intercept at zero (slope 1.01, Pearson correlation coefficient: 1.0), whereas the dashed red line displays the result of linear regression analysis of the non-sticky (blue) data with fixed intercept at zero (slope 0.07, Pearson correlation coefficient: 0.67). [file 12951_2018_426_MOESM6_ESM.pdf]

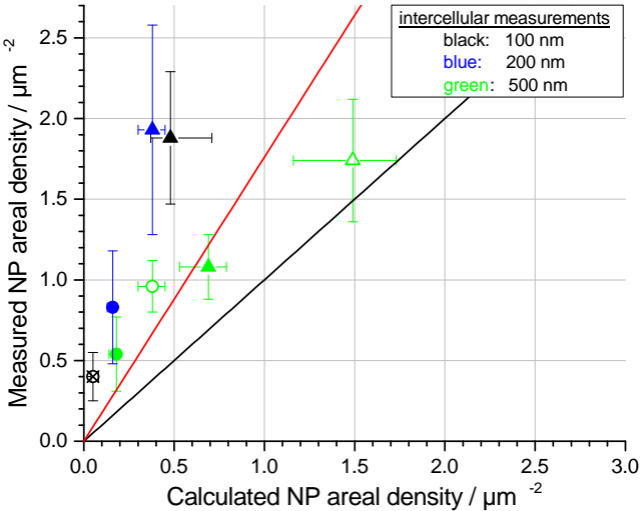

Supplement: Supplementary file 7 — Additional file 7. Measured intercellular ADs compared with calculated ADs using non-sticky boundary conditions. ITO/glass substrates covered with A549 cells were incubated with 100 nm (black), 200 nm (blue) and 500 nm (green) SiO2 particles at different concentrations for 1 h (circles) and 4 h (triangles). Full symbols denote 50 µg/mL input concentration, empty symbols 109 µg/mL and crossed symbols 7 µg/mL. The black diagonal line indicates an ideal match between measured and calculated ADs. The red line displays the result of linear regression analysis with fixed intercept at zero (slope 1.76, Pearson correlation coefficient: 0.87). Note the marked difference between the red and the black lines, indicating less agreement of measured and simulated results. [file 12951_2018_426_MOESM7_ESM.pdf]

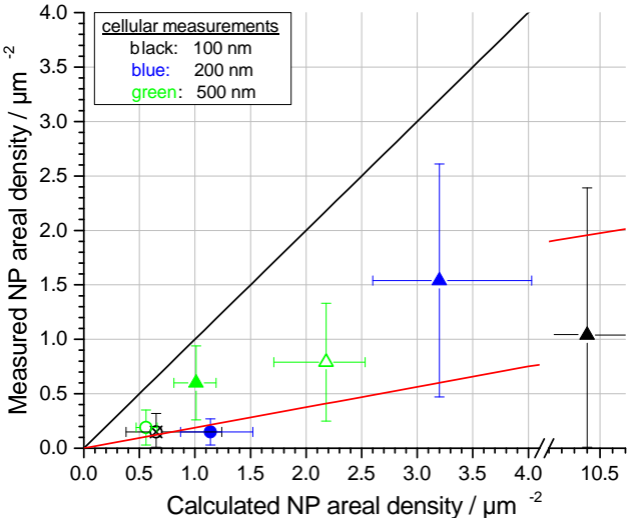

Supplement: Supplementary file 8 — Additional file 8. Measured cellular ADs compared with calculated ADs using sticky boundary conditions (KD = 10−9 mol/L). ITO/glass substrates covered with A549 cells were incubated with 100 nm (black), 200 nm (blue) and 500 nm (green) SiO2 particles at different concentrations for 1 h (circles) and 4 h (triangles). Full symbols denote 50 µg/mL input concentration, empty symbols 109 µg/mL and crossed symbols 7 µg/mL. The black diagonal line indicates an ideal match between measured and calculated ADs. The red line displays the result of linear regression analysis with fixed intercept at zero (slope 0.19, Pearson correlation coefficient: 0.82). Note the greatly differing slopes of the red and the black lines, indicating poor agreement of measured and simulated results. [file 12951_2018_426_MOESM8_ESM.pdf]

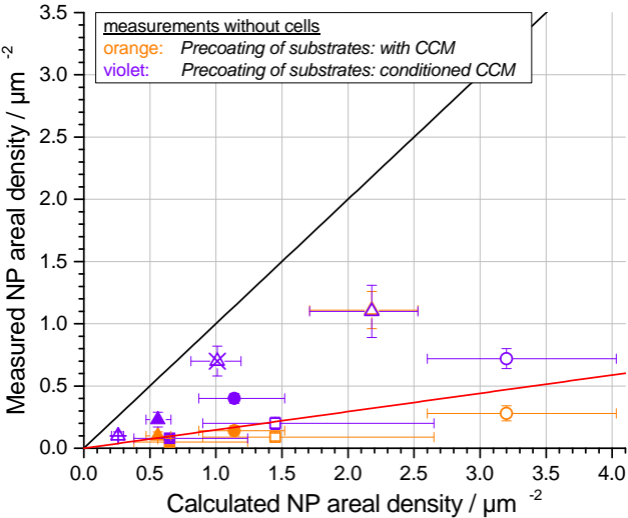

Supplement: Supplementary file 9 — Additional file 9. ADs measured on cell-free pre-coated substrates are compared with calculated ADs using sticky boundary conditions (KD = 10−9 mol/L). Deposition experiments were performed with cell-free ITO/glass substrates, precoated with CCM and conditioned CCM with 100 nm (squares), 200 nm (circles) and 500 nm (triangles) silica particles at different concentrations for 1 h (full symbols) and 4 h (empty symbols). Orange color represents pre-coatings performed with CCM and violet color represents pre-coatings with conditioned CCM. The black diagonal line indicates an ideal match between measured and calculated ADs. The red line displays the result of linear regression with fixed intercept at zero (slope 0.15, Pearson correlation coefficient: 0.8). Note the difference in slope between the red and the black lines, indicating poor agreement of measured and simulated results. [file 12951_2018_426_MOESM9_ESM.pdf]

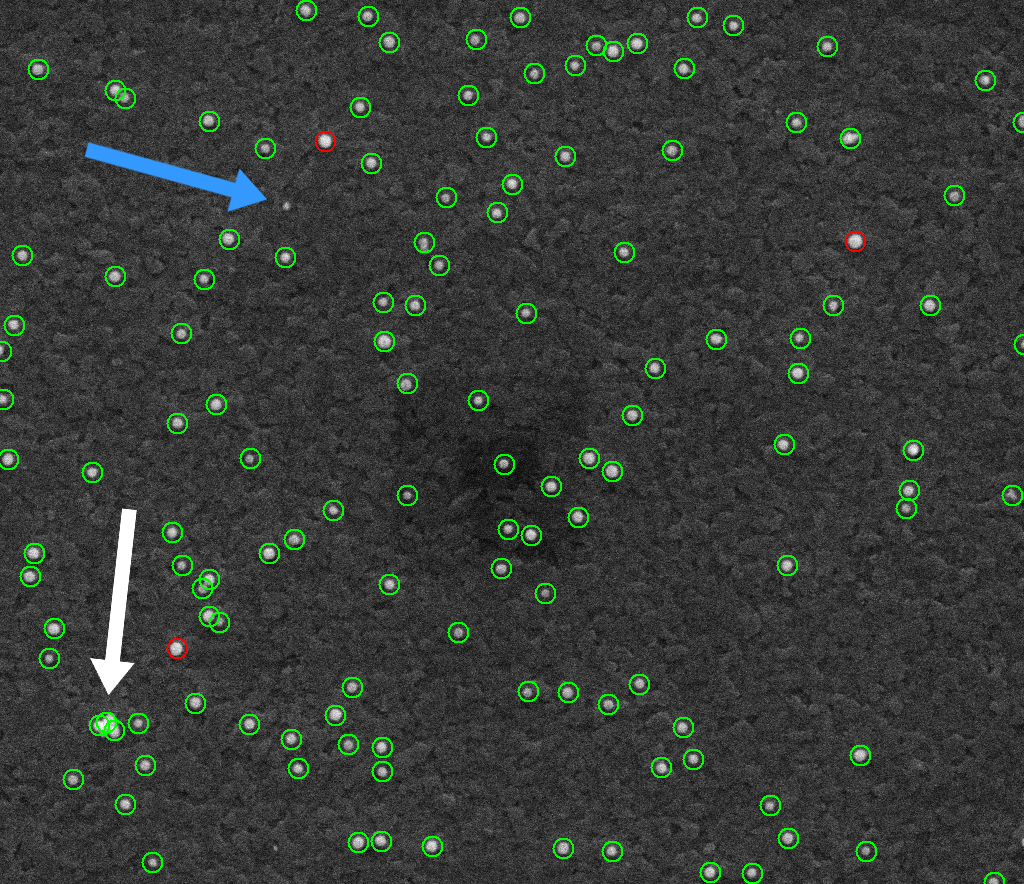

Supplement: Supplementary file 10 — Additional file 10. SE SEM image of 100 nm SiO2 NPs deposited on ITO substrate analysed with a semi-automated Matlab routine. The detected particles are counted and marked with colored circles, where green indicates accurate and red uncertain classification, which should be checked by the operator. The white arrow points at a small accumulation of NPs, of which only a small number has been detected, and the blue arrow shows a missed NP. After visual inspection, missing particles can be added, and false positives can be removed manually from the total sum of particles by the operator. [file 12951_2018_426_MOESM10_ESM.tif]
