# Supplementary material for: Assessment of in vitro particle dosimetry models at the single cell and particle level by scanning electron microscopy
Source: J Nanobiotechnology. 2018 Dec 7;16:100. doi: 10.1186/s12951-018-0426-2 (PMC6284276; doi:10.1186/s12951-018-0426-2)
Supplement: Supplementary file 4 — Additional file 4. Tabular comparison of calculated ADs to measured intercellular and cellular ADs after deposition of 100 nm, 200 nm and 500 nm SiO2 particles for 24 h. On ITO/glass substrates growing A549 cells were exposed to 100 nm 200 nm and 500 nm SiO2 particles for 24 h and then prepared for SEM analysis. Intercellular and cellular ADs were measured from SEM images by counting deposited particles. 12–24 regions of interest (ROI) were evaluated for each treatment. n.d.: not detectable. [file 12951_2018_426_MOESM4_ESM.docx]

| *SiO_2_ particle* | *Calculated NP areal density*  *[NP/µm^2^]* | *Measured NP areal density*  *[NP/µm^2^]* | |
| --- | --- | --- | --- |
|  |  | *intercellular* | *cellular* |
| *100 nm NP* | *0.7* | *0.007 ± 0.003* | *n.d.* |
| *200 nm MP* | *0.6* | *0.03 ± 0.03* | *n.d.* |
| *500 nm MP* | *0.6* | *0.77 ± 0.3* | *0.45 ± 0.2* |
