# Supplementary material for: Assessment of in vitro particle dosimetry models at the single cell and particle level by scanning electron microscopy
Source: J Nanobiotechnology. 2018 Dec 7;16:100. doi: 10.1186/s12951-018-0426-2 (PMC6284276; doi:10.1186/s12951-018-0426-2)
Supplement: Supplementary file 11 — Additional file 11. Tabular summary of the parameters used with the DG fate and transport model for computing particle deposition. [file 12951_2018_426_MOESM11_ESM.docx]

|  | parameter | value | units |
| --- | --- | --- | --- |
|  |  |  |  |
| solvent properties | solvent dynamic viscosity | 0.00074 | Pa s |
|  | density of solvent | 1.0 | g/cm³ |
|  | temperature of solvent | 37.0 | °C |
|  |  |  |  |
| particle properties | density of raw material | 2.0 | g/cm³ |
|  | diameters (dH) of particle / agglomerate species | 122 / 198 / 501 | nm |
|  | fraction of particle / agglomerate species | 1.0 |  |
|  | agglomerate effective density | n.a. | g/cm³ |
|  |  |  |  |
| experimental parameters | height of suspension column | 3.118 | mm |
|  | initial total concentration of material | Variable | mg/cm³ |
|  | total time of simulation | 1.0 / 4.0 | h |
|  | N x g (for centrifugation) | 1.0 |  |
|  |  |  |  |
| model parameters | height of subcompartment (simulation element) | 0.005 | mm |
|  | time interval for simulation | 0.5 | s |
|  |  |  |  |
| output data parameters | output data/graph report time interval | 1 | min |
|  | output compartment height | 0.01 | mm |
|  | plot / do not plot | 1 |  |
|  | bottom output only | 0 |  |
|  |  |  |  |
| advanced model parameters | sed. coeff. concentration dependence | 0.0 |  |
|  | diff. coeff. concentration dependence | 0.0 |  |
|  | initial dissolution fraction | 0.0 |  |
|  | method for modeling dynamic dissolution | 1 |  |
|  | type of dissolution rate | 0 |  |
|  | rate of dissolution | 0.048 |  |
|  | times for dissolution fraction data | [0.0, 12.0] | h |
|  | dissolution fractions corresponding to specified times | [0.0, 0.163 - 0.163] |  |
|  | stickiness | 0 / 1 |  |
|  | adsorption dissociation constant | 1.0E-09 |  |
